# Acrolein Exposure in U.S. Tobacco Smokers and Non-Tobacco Users: NHANES 2005–2006

**DOI:** 10.1289/ehp.1409251

**Published:** 2015-05-29

**Authors:** K. Udeni Alwis, B. Rey deCastro, John C. Morrow, Benjamin C. Blount

**Affiliations:** Tobacco and Volatiles Branch, Division of Laboratory Sciences, National Center for Environmental Health, Centers for Disease Control and Prevention, Atlanta, Georgia, USA

## Abstract

**Background:**

Acrolein is a highly reactive α,β unsaturated aldehyde and respiratory irritant. Acrolein is formed during combustion (e.g., burning tobacco or biomass), during high-temperature cooking of foods, and *in vivo* as a product of oxidative stress and polyamine metabolism. No biomonitoring reference data have been reported to characterize acrolein exposure for the U.S. population.

**Objectives:**

Our goals were to *a*) evaluate two acrolein metabolites in urine—*N*-acetyl-*S*-(3-hydroxypropyl)-l-cysteine (3HPMA) and *N*-acetyl-*S*-(2-carboxyethyl)-l-cysteine (CEMA)—as biomarkers of exposure to acrolein for the U.S. population by age, sex, race, and smoking status; and *b*) assess tobacco smoke as a predictor of acrolein exposure.

**Methods:**

We analyzed urine from National Health and Nutrition Examination Survey (NHANES 2005–2006) participants ≥ 12 years old (*n* = 2,866) for 3HPMA and CEMA using ultra-high-performance liquid chromatography coupled with electrospray ionization tandem mass spectrometry (UPLC/ESI-MSMS). Sample-weighted linear regression models stratified for non-tobacco users versus tobacco smokers (as defined by serum cotinine and self-report) characterized the association of urinary 3HPMA and CEMA with tobacco smoke exposure, adjusting for urinary creatinine, sex, age, and race/ethnicity.

**Results:**

3HPMA and CEMA levels were higher among tobacco smokers (cigarettes, cigars, and pipe users) than among non-tobacco users. The median 3HPMA levels for tobacco smokers and non-tobacco users were 1,089 and 219 μg/g creatinine, respectively. Similarly, median CEMA levels were 203 μg/g creatinine for tobacco smokers and 78.8 μg/g creatinine for non-tobacco users. Regression analysis showed that serum cotinine was a significant positive predictor (*p* < 0.0001) of both 3HPMA and CEMA among tobacco smokers.

**Conclusions:**

Tobacco smoke was a significant predictor of acrolein exposure in the U.S. population.

**Citation:**

Alwis KU, deCastro BR, Morrow JC, Blount BC. 2015. Acrolein exposure in U.S. tobacco smokers and non-tobacco users: NHANES 2005–2006. Environ Health Perspect 123:1302–1308; http://dx.doi.org/10.1289/ehp.1409251

## Introduction

Acrolein is a chemical contaminant that is ubiquitous in the environment. It is formed from carbohydrates, vegetable oils, animal fats, and amino acids during heating of foods and by combustion of petroleum fuels and biodiesel (Stevens and Maier 2008). Additionally, 1,3-butadiene can be photo-chemically degraded into acrolein in the environment (Doyle et al. 2004). Although there are a number of sources of exposure, smoking of tobacco products is typically the largest source of acrolein exposure (Stevens and Maier 2008). Consequently, the health impacts arising from the inhalation of acrolein are higher than those from other routes of exposure (Linhart et al. 1996). The amount of acrolein in cigarette smoke can vary from 18 to 98 μg per cigarette (Roemer et al. 2004). Carbohydrates, mainly sugar additives comprising 48–98 mg/g per cigarette (Wang and Watson 2012), are a major source of acrolein in cigarette smoke (Roemer et al. 2012; Stevens and Maier 2008). Acrolein is a toxic respiratory irritant that has been estimated to account for 97% of the total non-cancer respiratory hazard of mainstream cigarette smoke (Fowles and Dybing 2003). The United States Environmental Protection Agency (U.S. EPA) has determined that environmental exposure to acrolein is the leading cause of most non-cancer respiratory health effects (U.S. EPA 2002) at the national level. Furthermore, a recent study on acrolein exposure and asthma prevalence of the U.S. adult population (encompassing 2000–2009) estimated that chronic exposure to outdoor acrolein at concentrations of 0.05–0.46 μg/m^3^ was associated with an 8% increase in the prevalence-odds of having at least one asthma attack in the previous year (deCastro 2014).

Acrolein can also be formed endogenously as a product of polyamine metabolism and oxidative stress (Esterbauer et al. 1991; Igarashi and Kashiwagi 2011; Leung et al. 2011; Shi et al. 2011; Zhu et al. 2011). There is increasing evidence that endogenously formed acrolein is causally involved in physiological effects such as inflammation, atherosclerosis, cardiovascular and neurodegenerative diseases, and cancer (Guéraud et al. 2010; Lopachin et al. 2008; Tang et al. 2011; Wu et al. 2011). Levels of protein-conjugated acrolein in plasma and in the brain have been reported to be higher among subjects with mild cognitive impairment and Alzheimer’s disease than among age-matched normal control subjects (Bradley et al. 2010; Waragai et al. 2012). A recent study has observed an inverse correlation between brain infarction and urinary *N*-acetyl-*S*-(3-hydroxypropyl)-l-cysteine (3HPMA), supporting the idea that glutathione plays an important role in detoxification of acrolein at the cellular level (Tomitori et al. 2012; Yoshida et al. 2012). Feng et al. (2006) suggested that acrolein is a major etiological agent for cigarette smoke-related lung cancer and that it contributes to lung carcinogenesis by damaging DNA and inhibiting DNA repair.

During 2012–2013, an estimated 50 million people, or 19.2% of all adults (ages 18 years and older) in the United States, used some form of combustible tobacco product every day (72.1% of those used ≥ 1 combustible tobacco product daily) or some days [Centers for Disease Control and Prevention (CDC) 2014]. Cigarette smoking is the leading cause of preventable death in the United States, accounting for approximately 480,000 deaths, or 1 out of every 5 deaths, each year (DHHS 2014). Additionally, second- and third-hand smoke affects many U.S. residents, including a disproportionate number of children and infants (DHHS 2006, 2014; Pirkle et al. 2006; Protano et al. 2012). The tobacco product regulation group (TobReg) of the World Health Organization has identified acrolein as a major contributor to tobacco smoke toxicity (Burns et al. 2008).

**Figure 1:**
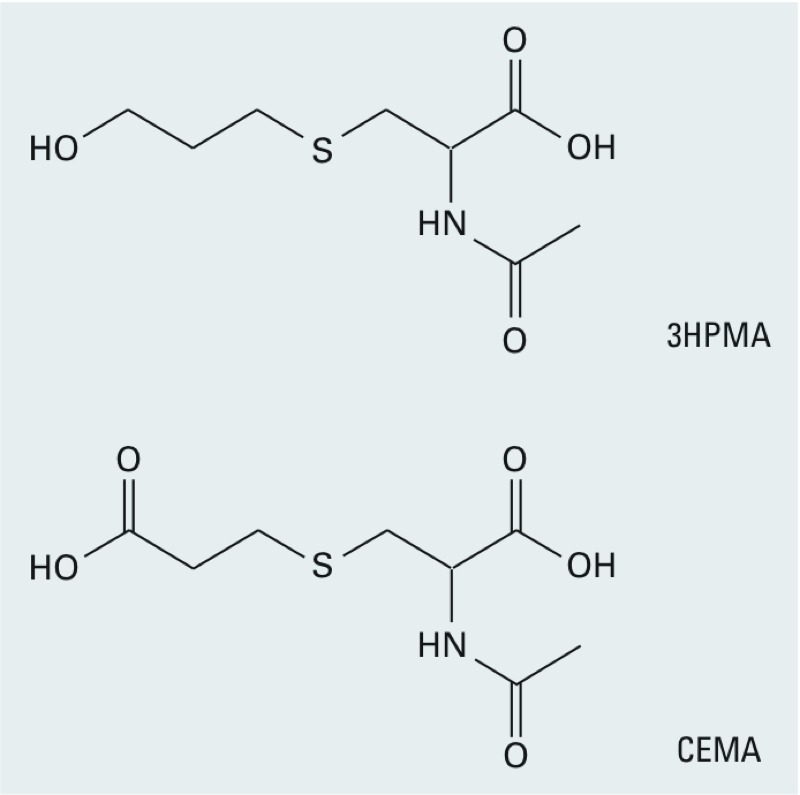
*N*-Acetyl-*S*-(3-hydroxypropyl)-l-cysteine (3HPMA) and *N*-acetyl-*S*-(2-carboxyethyl)-l-cysteine (CEMA) are urinary biomarkers of acrolein exposure.

3HPMA and *N*-acetyl-*S*-(2-carboxyethyl)-l-cysteine (CEMA) are specific urinary biomarkers of acrolein exposure (Figure 1). The main pathway by which acrolein is eliminated from the human body is through conjugation with glutathione (GSH) in the liver followed by enzymatic cleavage and *N*-acetylation to form *S*-(3-oxopropyl)-*N*-acetyl cysteine (OPMA) in the kidney (Kaye 1973). Reduction of the aldehyde group of OPMA forms 3HPMA, the major urinary metabolite of acrolein exposure, and oxidation of the aldehyde group of OPMA forms *N*-acetyl-*S*-[2-carboxyethyl]-l-cysteine (CEMA) as a minor metabolite.

Although a number of small-scale studies have been carried out to evaluate acrolein exposure (Carmella et al. 2007, 2009; Eckert et al. 2011; Schettgen et al. 2008), there have been no large-scale biomonitoring studies reported for the general population. Therefore, as part of the National Health and Nutrition Examination Survey (NHANES) 2005–2006, urine samples were assayed *a*) to evaluate 3HPMA and CEMA in urine as biomarkers of exposure to acrolein for the U.S. population by age, sex, race, and smoking status; and *b*) to assess tobacco smoke as a predictor of acrolein exposure.

## Methods

*Study design.* NHANES is a population-based survey designed to assess the health and nutrition status of a representative sample of the civilian, non-institutionalized population of the United States. NHANES is conducted by the National Center for Health Statistics (NCHS) of the Centers for Disease Control and Prevention (CDC). Spot urine samples were collected from a one-half subsample of NHANES 2005–2006 study participants ≥ 12 years old. The study protocol was reviewed and approved by the CDC institutional review board; additionally, written informed consent was obtained from all subjects before they took part in the study. The characteristics of the population in which the acrolein exposure was measured are given in Table 1.

**Table 1:** Sample-weighted demographic proportions of the NHANES 2005–2006 participants (sample sizes are unweighted).

Variable	NTU^*a*^		TS^*b*^	
	*n*^*c*^	Percent (SE)	*n*^*c*^	Percent (SE)
Age (years)
12–19	752	14.9 (0.91)	104	7.16 (0.93)
20–39	563	28.9 (1.52)	205	44.4 (3.48)
40–59	459	33.9 (1.56)	172	36.5 (3.32)
≥ 60	522	22.2 (2.22)	89	12.0 (1.70)
Race/ethnicity
Mexican American	628	9.41 (1.25)	78	4.88 (1.20)
Non-Hispanic black	558	10.3 (2.01)	178	13.5 (2.24)
Non-Hispanic white	942	71.1 (2.92)	275	73.8 (3.35)
Other Hispanic or other/multi-race	168	9.18 (1.51)	39	7.83 (1.85)
Sex
Female	1,288	54.9 (0.97)	248	45.7 (2.00)
Male	1,008	45.1 (0.97)	322	54.3 (2.00)
^***a***^Non-tobacco users (NTU): defined for this study as non-smokers with serum cotinine ≤ 10 ng/mL. ^***b***^Tobacco smokers (TS): defined for this study as smokers with serum cotinine > 10 ng/mL, using cigarettes, cigars, or pipes during the last 5 days prior to physical examination. ^***c***^Sample size.				

*Analytical method.* In the current study, residual spot urine samples from NHANES 2005–2006 that were stored at –70 °C for 4–5 years were analyzed for 3HPMA and CEMA. An aliquot of 100 μL of each sample was assayed for 28 volatile organic metabolites using ultra-high-performance liquid chromatography (Waters Inc., Milford, MA) coupled with electrospray tandem mass spectrometry (UPLC/ESI-MSMS; Sciex API 4000 Triple Quad; Applied Biosystems, Foster City, CA) (Alwis et al. 2012). Urine samples were assayed at 1:10 dilution (100 μL urine + 50 μL working mixed internal standard + 850 μL 15 mM ammonium acetate). A mixture of 28 internal standards labeled with stable isotopes was used to quantify the levels. Chromatographic separation was achieved using an Acquity UPLC® HSS T3 1.8 μm × 2.1 mm × 150 mm (Waters Inc.) column with 15 mM ammonium acetate, pH 6.8 (Solvent A) and acetonitrile (Solvent B) as mobile phases. The eluent from the column was ionized using an electrospray interface to generate and transmit negative ions into the mass spectrometer. Comparison of relative response factors (ratio of native analyte to stable-isotope-labeled internal standard) with known standard concentrations yielded individual analyte concentrations for unknowns. Analyst software (version 1.5.1; Applied Biosystems, Foster City, CA) was used to operate both the UPLC and the API4000. The mass spectrometer was operated in scheduled multiple reaction monitoring (SMRM) mode for negative ions, the ion source temperature was kept at 650°C, and the electrospray ion voltage was kept at –4,000 V. The mass parameters were optimized for each analyte. For 3HPMA, *m/z* 220/91 and *m/z* 220/89 were monitored as quantitation and confirmation ion transitions, respectively. For CEMA, *m/z* 234/162 (quantitation ion transition) and *m/z* 234/105 (confirmation ion transition) were monitored. We monitored *m/z* 226/97 for ^2^H_6__3HPMA and *m/z* 237/162 for ^13^C_3__CEMA. The limits of detection (LODs) for 3HPMA and CEMA were 13 and 8 ng/mL, respectively.

*Statistics.* The NCHS has conducted NHANES since the early 1960s to assess the health and nutritional status of the United States through the collection of serial cross-sectional data from a complex, multistage probability sample representative of the civilian, non-institutionalized population of the United States (CDC 2005–2006). 3HPMA and CEMA were measured in single spot urine samples obtained from participants ≥ 12 years old during physical examinations performed in mobile examination centers. Reported results met the accuracy and precision specifications of the quality control/quality assurance program of the CDC National Center for Environmental Health, Division of Laboratory Sciences (Caudill et al. 2008). Measurements below the LOD were substituted with the quotient of the LOD divided by the square root of two.

The acrolein biomarkers 3HPMA and CEMA were measured in urine collected from 3,545 NHANES participants. Acrolein is present in tobacco smoke; therefore, NHANES participants who recently smoked tobacco were distinguished from non-tobacco users when serum cotinine (a nicotine-specific biomarker, measured in a serum sample obtained at the same time as the spot urine sample) exceeded 10 ng/mL (Pirkle et al. 1996). A total of 271 participants were excluded from the analytical data set because they lacked serum cotinine data. In addition, to focus the analysis on combustible–tobacco product smokers (called “tobacco smokers” throughout the rest of this report), participants with serum cotinine exceeding 10 ng/mL were included in the analytical dataset if they also responded “yes” to NHANES smoking questionnaire question SMQ680 (tobacco or nicotine use within 5 days prior to NHANES physical examination), responded “yes” to at least one of questions SMQ690A–SMQ690C (cigarettes, pipes, cigars), and responded “no” to all of questions SMQ690D–SMQ690F (smokeless tobacco and nicotine delivery products). Non-tobacco users had serum cotinine ≤ 10 ng/mL and answered “no” to SMQ680. Another 172 participants were excluded for not having answered SMQ680, and a further 206 participants were excluded because data were missing for other variables used in the regression models, leaving 2,866 participants to be included in the statistical analysis.

Because NHANES participants are recruited through a multistage sampling design, it is necessary to account for this complex design in order to properly estimate variances and to produce unbiased, nationally representative statistics. Robust estimation may be accomplished through a generalized estimation equations approach incorporating Taylor series linearization and applying survey sample weights to each survey participant. We used this estimation approach as it was implemented in the DESCRIPT and REGRESS subroutines of SUDAAN version 11.0.0 (Research Triangle Institute, Research Triangle Park, NC) for SAS version 9.3 statistical software (SAS Institute Inc., Cary, NC). Sample-weighted linear regression models stratified by tobacco use status (tobacco smokers vs. non-tobacco users) were fit to NHANES data from the 2005–2006 survey cycles (CDC 2005–2006) where the dependent variable was the urinary concentration of 3HPMA or CEMA (micrograms per liter). Because the distribution of urinary measurements was highly right skewed, which would have adversely affected hypothesis testing, the urinary concentration data were natural-log transformed in order to evaluate the statistical significance of the regression slopes. The *p*-values for slopes from the ln urinary concentration regression models are reported herein. To facilitate interpretability, however, we have reported the slopes and their 95% confidence intervals estimated from identical regression models of untransformed urinary concentration data. Statistical significance was set at α ≤ 0.05, and marginal significance was set at 0.05 < α ≤ 0.15.

Additional self-reported predictors included in the regression models were sex, age, and race/ethnicity. Age (years) was divided into the following ranges: 12–19, 20–39, 40–59, and ≥ 60. Serum cotinine was used as a continuous variable to evaluate the association between urinary concentration of 3HPMA and CEMA and tobacco smoke exposure in the regression model for both tobacco smokers and non-tobacco users. Among non-tobacco users, tobacco smoke exposure is primarily attributable to secondhand smoke (SHS), which is associated with serum cotinine. In order to directly associate urinary biomarker concentrations with the frequency of cigarette smoking, we ran the same regression model for participants who reported exclusive use of cigarettes, but we replaced serum cotinine with self-reported average number of cigarettes smoked per day (CPD) over the 5 days preceding the mobile examination center (MEC) exam, classified in ranges of 1–10 CPD (0.5 pack), 11–20 (1 pack), 21–30 (1.5 packs), and > 30 (> 1.5 packs), where the reference category comprised self-reported non-tobacco users with serum cotinine ≤ LOD (0.015 ng/mL).

3HPMA and CEMA, respectively, are major and minor metabolites of acrolein exposure. Both are formed from the same intermediate, *S*-(3-oxopropyl)-*N*-acetyl cysteine (OPMA). Smoking can elevate levels of 3HPMA and CEMA. Furthermore, enzyme activity metabolizing acrolein to 3HPMA and CEMA can vary among races. To investigate differences in acrolein metabolism according to race, we calculated sample-weighted CEMA/3HPMA molar ratios by smoking status and race/ethnicity for the NHANES 2005–2006 population.

Because urinary biomarker concentrations can be influenced by urine dilution, which can vary markedly throughout the day within each individual, statistical inference can be confounded (Barr et al. 2005). Urine dilution can be accounted for by scaling urinary analyte concentration to the urinary concentration of creatinine, a compound that is excreted endogenously at a fairly constant rate and is therefore resistant to urine dilution. Summary statistics for urine 3HPMA and CEMA according to demographic characteristics and tobacco use have been reported as the urinary concentration ratio of 3HPMA and CEMA to creatinine (μg/g creatinine), whereas the regression models used to estimate the associations between the urinary concentrations of 3HPMA and CEMA included urinary creatinine (grams per milliliter) as a covariate to account for urine dilution.

## Results

3HPMA and CEMA were detected in 99 and 98%, respectively, of the samples assayed. Urinary 3HPMA and CEMA levels were higher among tobacco smokers than non-tobacco users (*p* < 0.0001) for the NHANES 2005–2006 population (Table 2; for geometric means and percentiles, see Supplemental Material, Tables and S2). Our multiple regression model of urinary 3HPMA among tobacco smokers (Table 3), adjusted for sex, age, and race/ethnicity, showed that serum cotinine was positively associated with 3HPMA levels in urine (*p* < 0.0001). 3HPMA levels were significantly lower in 12- to 19- and 20- to 39-year-olds than in 40- to 59-year-olds; additionally, Mexican Americans, non-Hispanic blacks, and other Hispanics or other/multi-race had significantly lower levels of 3HPMA than non-Hispanic whites. A multiple regression model for urinary CEMA among tobacco smokers (Table 3) showed similar results: serum cotinine was positively associated with CEMA levels in urine (*p* < 0.0001); CEMA levels were significantly lower among 12- to 19- and 20- to 39-year-olds compared with 40- to 59-year-olds; and Mexican Americans had significantly lower CEMA levels than non-Hispanic whites. In contrast with 3HPMA, there were no significant differences in CEMA levels when comparing non-Hispanic blacks and other/multi-race with non-Hispanic whites. Among non-tobacco users, our regression model data showed that serum cotinine was a significant predictor of CEMA (*p* = 0.007) but not 3HPMA (*p* = 0.8) (see Supplemental Material, Table S3).

**Table 2:** Sample-weighted median (25th, 75th percentile) urinary 3HPMA and CEMA concentrations (creatinine adjusted) by age, sex, and race/ethnicity categorized by smoking status among NHANES 2005–2006 participants ≥ 12 years old (sample sizes are unweighted).

Variable	Sample size (*n*)		3HPMA [μg/g creatinine (25th, 75th percentile)]		CEMA [μg/g creatinine (25th, 75th percentile)]	
	NTU	TS	NTU	TS	NTU	TS
All	2,467	601	219 (140, 353)	1,089 (469, 2,012)	78.8 (51.8, 121)	203 (111, 338)
Age (years)
12–19	811	114	192 (130, 285)	477 (333, 755)	65.5 (44.5, 103)	122 (83.3, 202)
20–39	618	208	216 (136, 348)	836 (430, 1,641)	71.8 (49.0, 104)	157 (97.1, 253)
40–59 (reference)	493	177	239 (146, 400)	1,602 (691, 2,714)	82.2 (50.8, 130)	246 (142, 407)
≥ 60	545	102	215 (140, 332)	1,375 (702, 2,345)	94.2 (62.8, 146)	309 (170, 415)
Race/ethnicity
Mexican American	685	82	243 (154, 410)	445 (254, 843)	78.3 (52.4, 121)	102 (69.6, 171)
Non-Hispanic black	613	191	199 (128, 299)	741 (409, 1,342)	81.9 (53.9, 130)	191 (111, 297)
Non-Hispanic white (reference)	984	287	216 (137, 345)	1,248 (519, 2,255)	78.4 (51.2, 119)	212 (119, 346)
Other Hispanic or other/multi-race	185	41	261 (175, 400)	1,094 (370, 1,687)	80.8 (53.7, 122)	178 (104, 347)
Sex
Female (reference)	1,379	252	211 (132, 369)	1,269 (579, 2,248)	80.5 (50.7, 127)	233 (116, 378)
Male	1,088	349	233 (148, 344)	932 (432, 1,796)	77.8 (53.1, 116)	178 (108, 298)
Abbreviations: NTU, non-tobacco users; TS, tobacco smokers.						

**Table 3:** Sample-weighted multiple regression slopes for urinary 3HPMA and CEMA concentrations among NHANES 2005–2006 tobacco smokers.

Variable	3HPMA		CEMA	
	Coefficient (95% CI)	*p-*Value	Coefficient (95% CI)	*p-*Value
Intercept	–90.8 (–692, 510)	< 0.0001	–12.4 (–60.5, 35.7)	< 0.0001
Serum cotinine (ng/mL)	5.05 (3.35, 6.74)	< 0.0001	0.63 (0.41, 0.84)	< 0.0001
Creatinine (g/mL)	1,117,490 (757,159, 1,477,821)	< 0.0001	200,849 (150,890, 250,808)	< 0.0001
Age (years)
12–19	–947 (–1,358, –536)	0.002	–117 (–205, –28.7)	0.01
20–39	–658 (–965, –350)	0.006	–112 (–179, –44.6)	0.001
40–59	Reference		Reference
≥ 60	–318 (–773, 138)	0.23	26.1 (–31.4, 83.7)	0.88
Sex
Female	Reference		Reference
Male	–189 (–391, 13.3)	0.69	–25.5 (–74.5, 23.5)	0.79
Race/ethnicity
Mexican American	–556 (–991, –121)	0.003	–74.1 (–125, –23.3)	0.0006
Non-Hispanic black	–902 (–1,188, –615)	< 0.0001	–20.7 (–98.5, 57.1)	0.67
Non-Hispanic white	Reference		Reference
Other Hispanic or other/multi-race	–751 (–1,316, –186)	0.02	–70.1 (–169, 29.1)	0.29
*p*-Value was estimated from identical models where the dependent variable was natural log-transformed.				

**Figure 2:**
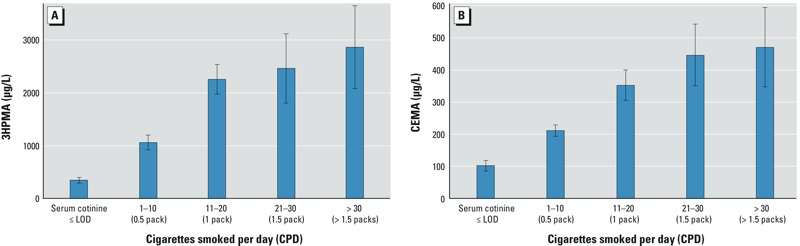
Least-square mean (95% confidence intervals) for each cigarette smoked per day (CPD) category of urinary 3HPMA (*A*) and CEMA (*B*) concentrations among exclusive cigarette smokers with serum cotinine > 10 ng/mL, adjusted for sex, age, race/ethnicity, and urinary creatinine.

The associations between urinary biomarkers and CPD for exclusive cigarette smokers were also examined for the same population. Figure 2 displays the least-square mean of the natural log of urinary 3HPMA and CEMA concentrations for each CPD category adjusted for sex, age, race/ethnicity, and urinary creatinine. Both 3HPMA and CEMA levels increased with increasing values of CPD among exclusive cigarette smokers. The multiple regression models of urinary 3HPMA and CEMA among exclusive cigarette smokers, adjusted for sex, age, and race/ethnicity (see Supplemental Material, Table S4), also showed that both urinary 3HPMA and CEMA increased in a dose-dependent manner across participants who smoked 1–10, 11–20, 21–30, and > 30 CPD compared to those with serum cotinine ≤ LOD. Among age groups, 3HPMA levels were significantly lower for 12- to 19-, 20- to 39-, and ≥ 60-year-olds than for 40- to 59-year-olds. CEMA levels were significantly lower for 12- to 19- and 20- to 39-year-olds than for 40- to 59-year-olds. According to NHANES 2005–2006 demographic information, among smokers (serum cotinine > 10 ng/mL), 43% of the participants were in the 1–10 CPD (0.5 pack) category, 35% in the 11–20 CPD (1 pack) category, 9% in the 21–30 CPD (1.5 packs) category, and 6% in the > 30 CPD (> 1.5 packs) category.

The CEMA/3HPMA molar ratio was significantly higher for non-tobacco users than for tobacco smokers (*p* < 0.001) and varied among race/ethnic groups within smoking- status categories (Table 4). Non-Hispanic blacks had significantly higher CEMA/3HPMA ratios for both tobacco smokers and non-tobacco users than non-Hispanic whites. Among non-tobacco users, Mexican Americans and other Hispanic or other/multi-race participants had significantly lower CEMA/3HPMA ratios than non-Hispanic whites (*p* = 0.003 and 0.03, respectively), and among tobacco smokers, other Hispanic or other/multi-race participants had significantly higher CEMA/3HPMA ratios than non-Hispanic whites (*p* = 0.03).

**Table 4:** Sample-weighted molar ratios of urinary acrolein metabolites (CEMA/3HPMA) by smoking status and race/ethnicity.

Variable	GM (95% CI)	Median (25th, 75th percentile)	*p-*Value
Race/ethnicity
Tobacco smokers (TS)
Mexican American	0.20 (0.14, 0.26)	0.21 (0.14, 0.33)	0.18
Non-Hispanic black	0.24 (0.22, 0.26)	0.23 (0.16, 0.36)	0.0001
Non-Hispanic white	0.17 (0.15, 0.18)	0.17 (0.12, 0.25)	Reference
Other Hispanic or other/multi-race	0.21 (0.16, 0.26)	0.20 (0.13, 0.31)	0.03
Non-tobacco users (NTU)
Mexican American	0.27 (0.24, 0.30)	0.28 (0.18, 0.46)	0.003
Non-Hispanic black	0.38 (0.36, 0.41)	0.39 (0.25, 0.59)	0.004
Non-Hispanic white	0.33 (0.31, 0.35)	0.34 (0.21, 0.52)	Reference
Other Hispanic or other/multi-race	0.28 (0.25, 0.32)	0.28 (0.18, 0.46)	0.03
Serum cotinine
≤ 10 ng/mL	0.32 (0.31, 0.34)	0.33 (0.21, 0.52)	0.0001
> 10 ng/mL	0.18 (0.17, 0.20)	0.18 (0.13, 0.29)	Reference
GM, geometric mean.			

**Figure 3:**
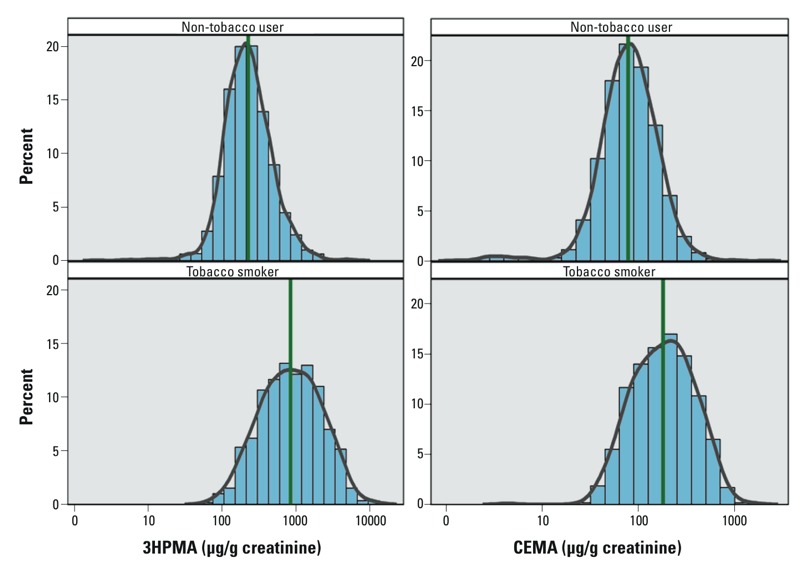
Percent distribution of urinary 3HPMA and CEMA (μg/g creatinine) among tobacco smokers and non-tobacco users: NHANES 2005–2006 (percentages not sample weighted).

We used serum cotinine to distinguish tobacco smokers from non-tobacco users in the NHANES 2005–2006 sample population. In unweighted analysis, significant correlations (*p* < 0.0001) were found between serum cotinine and urinary acrolein biomarkers for tobacco smokers (*r* = 0.52 for 3HPMA and serum cotinine; *r* = 0.45 CEMA and serum cotinine). The unweighted correlation between 3HPMA and CEMA was strong among tobacco smokers (*r* = 0.77, *p* < 0.0001) compared to non-tobacco users (*r* = 0.46, *p* < 0.0001). The percent distribution of urinary 3HPMA and CEMA among tobacco smokers and non-tobacco users for the U.S. population is shown in Figure 3.

## Discussion

Acrolein is a reactive aldehyde that is also a toxic respiratory irritant, cardiotoxicant, and carcinogen. Among exogenous sources, tobacco smoke is a major source of exposure to acrolein (Stevens and Maier 2008, 2011). The human body detoxifies acrolein by conjugating it with glutathione, producing 3HPMA and CEMA as major and minor byproducts, respectively (Stevens and Maier 2011). Consistent with the published literature, we observed that among tobacco smokers, the median measured 3HPMA level (1,089 μg/g creatinine) was five times greater than the median CEMA level (203 μg/g creatinine). The significant associations found between urinary 3HPMA and CEMA with serum cotinine among tobacco smokers, and the dose-dependent relationships between biomarkers with CPD among exclusive cigarette smokers, show that 3HPMA and CEMA are effective urinary biomarkers of tobacco smoke–related acrolein exposure. The levels of these biomarkers were significantly higher among tobacco smokers than non-tobacco users.

3HPMA and CEMA are formed from the intermediate OPMA [*S*-(3-oxopropyl)-*N*-acetyl cysteine]. Reduction of the aldehyde group of OPMA by an aldo-keto reductase forms 3HPMA, whereas CEMA is formed via oxidation of the aldehyde group of OPMA by an aldehyde dehydrogenase. Because compounds are formed from the same intermediate, a significant difference in the CEMA/3HPMA molar ratio among tobacco smokers and non-tobacco users may be expected. 3HPMA is the major urinary metabolite of acrolein exposure (Stevens and Maier 2008), and in this study population, the urinary 3HPMA levels were five times higher than the urinary CEMA levels in tobacco smokers. Among non-tobacco users, 3HPMA levels were approximately three times higher than CEMA levels. Therefore, the CEMA/3HPMA ratio was significantly higher (*p* < 0.0001) in non-tobacco users (0.32) than in tobacco smokers (0.18). We also found a significantly higher CEMA/3HPMA ratio for non-Hispanic blacks than for non-Hispanic whites. Currently, there is no information in the literature about the CEMA/3HPMA ratio or variations in the enzymatic metabolization of acrolein to mercapturic acids among races. Our findings may help guide future research to understand the phase II detoxification of acrolein by enzymes, the inhibitors and inducers of enzyme activity, and variations of acrolein-metabolizing enzyme activity among different races.

The measured 3HPMA levels in our study were approximately four times higher in tobacco smokers than in non-tobacco users, consistent with all but one previous study (Schettgen et al. 2008) (Table 5). Our study found median urinary 3HPMA levels of 219 and 1,089 μg/g creatinine for non-tobacco users and tobacco smokers, respectively. A European study (Eckert et al. 2011) reported similar median values: 146 and 884 μg/g creatinine, respectively, for non-tobacco users and tobacco smokers. Similar mean 3HPMA levels were observed for tobacco smokers and non-tobacco users in two large-scale studies [the present study and Roethig et al. (2009)] investigating volatile organic compound exposure among the U.S. population (Table 5).

**Table 5:** Comparison of urinary biomarker levels among tobacco smokers and non-tobacco users.

Source	No. of subjects		3HPMA		CEMA	
	NTU	TS	NTU	TS	NTU	TS
Mean creatinine (μg/g)
Current study	2,467	600	309	1,510	99.4	248
Roethig et al. 2009^*a*^	1,077	3,585	327	1,450
Scherer et al. 2007^*b*^	100	194	241	926
Mascher et al. 2001^*c*^	41	27	580	2,006
Median creatinine (μg/g)
Current study	2,467	600	219	1,089	78.8	203
Eckert et al. 2011^*d*^	54	40	146	884
Schettgen et al. 2008^*e*^	14	14	113	1,630
Carmella et al. 2007^*f*^	21	35	151	643
^***a***^Smokers were defined as people who regularly consumed a minimum of one manufactured cigarette per day over the last 12 months. Nonsmokers were defined as people who did not use tobacco or nicotine-containing products during the 5 years prior to the study. ^***b***^Smokers were self-reported daily cigarette users; cotinine and *trans*-3′-hydroxycotinine were analyzed in saliva of both smokers and nonsmokers; nicotine equivalents and 4-(methylnitrosamino)-1-(3-pyridyl)-1-butanol (NNAL) were analyzed in smoker urine samples as part of the study. ^***c***^Self-reported. ^***d***^Smoking status was verified by urine cotinine analysis in addition to self-reported smoking behavior: Smoker urinary cotinine levels ranged from 0.7 to 136 μg/L, and nonsmoker levels ranged from 52.4 to 3,752 μg/L. ^***e***^Smoking status was verified by urine cotinine analysis in addition to self-reported smoking behavior: Smoker urinary cotinine levels ranged from 713 to 3,073 μg/L, and nonsmoker levels ranged from < 1 to 22 μg/L. ^***f***^Self-reported.						

## Conclusions

The NHANES 2005–2006 3HPMA and CEMA data provide the first reference levels of acrolein exposure for the U.S. population against which subsequent NHANES and other large scale epidemiologic studies may be compared. Levels of the urinary biomarkers 3HPMA and CEMA characterize acrolein exposure in the U.S. population. Acrolein, a combustion product in tobacco smoke, has been estimated to account for 97% of the total non-cancer respiratory hazard of mainstream cigarette smoke (Fowles and Dybing 2003). Although the prevalence of cigarette smoking among U.S. adults in 2012–2013 (18%) was significantly lower than that observed in 2009–2010 (19.5%), cigarettes and other combustible products remained the most prevalent forms of adult tobacco use in the United States (CDC 2014). Future efforts will explore acrolein exposure from other sources (e.g., acrolein in indoor and outdoor air), investigate the association between acrolein exposure and health effects, and evaluate the efficacy of strategies to reduce the levels of acrolein in major sources such as tobacco smoke.

## Supplemental Material

(271 KB) PDF
