# Supplementary material for: Acrolein Exposure in U.S. Tobacco Smokers and Non-Tobacco Users: NHANES 2005–2006
Source: Environ Health Perspect. 2015 May 29;123(12):1302–8. doi: 10.1289/ehp.1409251 (PMC4671235; doi:10.1289/ehp.1409251)
Supplement: (271 KB) PDF [file ehp.1409251.s001.acco.pdf]

Environ Health Perspect
DOI: 10.1289/ehp.1409251

Note to Readers: *EHP* strives to ensure that all journal content is accessible to all readers. However, some figures and Supplemental Material published in *EHP* articles may not conform to 508 standards due to the complexity of the information being presented. If you need assistance accessing journal content, please contact ehp508@niehs.nih.gov. Our staff will work with you to assess and meet your accessibility needs within 3 working days.

Supplemental Material

Acrolein Exposure in U.S. Tobacco Smokers and Non-Tobacco Users: NHANES 2005-2006

K. Udeni Alwis, B. Rey deCastro, John C. Morrow, and Benjamin C. Blount

Table of Contents

Table S1. Sample-weighted median (25th, 75th percentile) urinary 3HPMA concentration µg/g creatinine by age, sex, and race/ethnicity among NHANES 2005-2006 tobacco smokers and non-tobacco users (sample size unweighted).

Table S2. Sample-weighted median (25th, 75th percentile) urinary CEMA concentration µg/g creatinine by age, sex, and race/ethnicity among NHANES 2005-2006 tobacco smokers and non-tobacco users (sample size unweighted).

Table S3. Sample-weighted multiple regression slopes for urinary 3HPMA and CEMA concentrations (µg/L) among NHANES 2005-2006 non-tobacco users.

Table S4. Sample-weighted multiple regression slopes for urinary 3HPMA and CEMA concentrations (µg/L) among NHANES 2005-2006 exclusive cigarette smokers with serum cotinine > 10 ng/mL.

Table S1. Sample-weighted median (25th, 75th percentile) urinary 3HPMA concentration μg/g creatinine by age, sex, and race/ethnicity among NHANES 2005-2006 tobacco smokers and non-tobacco users (sample size unweighted).

Variable	GM (95% CI)[a]	Median (25th, 75th percentile)	Percentiles						n[b]
			2.5th	5th	10th	90th	95th	97.5th	
**Tobacco smokers (TS)**									
All	990 (854, 1126)	1089 (469, 2012)	144	194	284	3415	4225	5081	600
Age (yr)									
12 - 19	520 (362, 677)	477 (333, 755)	135	143	165	1469	2623	2824	114
20 - 39	813 (682, 944)	836 (430, 1641)	173	189	242	2287	3024	3621	208
40 - 59 [Ref]	1324 (1053, 1594)	1602 (691, 2714)	122	201	310	3952	4954	5577	177
≥ 60	1191(813, 1570)	1375 (702, 2345)	174	225	276	3789	4488	5094	102
Race/ethnicity									
Mexican American	499 (396, 602)	445 (254, 843)	110	164	194	1431	2459	3782	82
Non-Hispanic Black	747 (630, 864)	741 (409, 1342)	170	195	234	1928	2371	3464	191
Non-Hispanic White [Ref]	1108 (932, 1284)	1248 (519, 2255)	144	194	315	3634	4499	5409	287
Other Hispanic or Other/multi race	854 (627, 1083)	1094 (370, 1687)	.	117	292	2526	3127	3516	41
Sex									
Female [Ref]	1127 (1003, 1252)	1269 (579, 2248)	180	206	316	3417	4423	5719	252
Male	893 (713, 1072)	932 (432, 1796)	140	174	242	3155	3978	4614	349
**Non-tobacco users (NTU)**									
All	227 (212, 242)	219 (140, 353)	57.5	76.2	99.7	562	833	1112	2,467
Age (yr)									
12 - 19	197 (182, 213)	192 (130, 285)	57.4	68.4	97.1	426	540	754	811
20 - 39	226 (198, 253)	216 (136, 348)	37.2	76.5	93.9	593	833	1125	618
40 - 59 [Ref]	247 (228, 266)	239 (146, 400)	64.8	76.7	106	622	900	1156	493
≥ 60	221 (204, 238)	215 (140, 332)	57.4	71.4	99.2	546	761	986	545
Race/ethnicity									
Mexican American	253 (231, 275)	243 (154, 410)	74.7	86.7	104	661	909	1191	685
Non-Hispanic Black	198 (176, 221)	199 (128, 299)	49.9	72.3	88.1	461	695	962	613
Non-Hispanic White [Ref]	224 (207, 241)	216 (137, 345)	55.7	74.8	98.9	584	834	1108	984
Other Hispanic or Other/multi race	260 (218, 303)	261 (175, 400)	68.6	82.9	119	504	762	1004	185
Sex									
Female [Ref]	225 (206, 243)	211 (132, 369)	55.6	72.2	92.2	639	889	1353	1,379
Male	230 (214, 245)	233 (148, 344)	65.9	83.8	111	505	702	946	1,088

[a]Geometric mean (95% confidence intervals). [b]Sample size.

Table S2. Sample-weighted median (25th, 75th percentile) urinary CEMA concentration µg/g creatinine by age, sex, and race/ethnicity among NHANES 2005-2006 tobacco smokers and non-tobacco users (sample size unweighted).

Variable	GM (95% CI)[a]	Median (25th, 75th percentile)				Percentiles			n[b]
			2.5th	5th	10th	90th	95th	97.5th	
**Tobacco smokers (TS)**									
All	191 (172, 209)	203 (111, 338)	41.4	49.6	67.7	500	588	685	601
Age (yr)									
12 - 19	134 (105, 164)	122 (83.3, 202)	51.0	53.8	59.9	304	361	466	114
20 - 39	156 (136, 175)	157 (97.1, 253)	43.6	49.4	63.4	386	472	558	208
40 - 59 [Ref]	232 (198, 266)	246 (142, 407)	37.7	44.7	78.6	558	655	728	177
≥ 60	262 (199, 324)	309 (170, 416)	37.9	71.3	84.4	538	634	769	102
Race/ethnicity									
Mexican American	107 (82.0, 132)	102 (69.6, 171)	11.4	37.2	43.4	252	434	466	82
Non-Hispanic Black	189 (161, 217)	191 (111, 296)	57.7	63.9	79.4	439	607	654	191
Non-Hispanic White [Ref]	198 (174, 223)	212 (119, 346)	39.4	48.1	67.2	504	609	690	287
Other Hispanic or Other/multi race	192 (141, 244)	178 (104, 347)	53.3	55.0	79.4	504	548	549	41
Sex									
Female [Ref]	211 (190, 232)	233 (116, 378)	45.1	49.6	76.5	520	580	710	252
Male	176 (152, 201)	179 (108, 298)	38.6	48.9	65.0	446	596	652	349
**Non-tobacco users (NTU)**									
All	77.7 (72.9, 82.4)	78.8 (51.8, 121)	18.8	26.8	35.3	183	228	296	2,467
Age (yr)									
12 - 19	67.2 (59.3, 75.1)	65.5 (44.5, 103)	20.0	26.1	33.7	158	192	217	811
20 - 39	68.9 (62.3, 75.4)	71.8 (49.0, 104)	14.7	25.3	32.6	154	205	247	618
40 - 59 [Ref]	79.8 (73.2, 86.4)	82.2 (50.8, 130)	16.9	25.8	34.6	198	236	294	493
≥ 60	96.5 (89.4, 104)	94.2 (62.8, 146)	27.9	37.9	45.4	209	288	362	545
Race/ethnicity									
Mexican American	72.7 (67.3, 78.0)	78.3 (52.4, 121)	4.27	16.5	34.7	172	211	244	685
Non-Hispanic Black	80.8 (72.2, 89.3)	81.9 (53.9, 130)	19.7	26.9	35.4	185	261	304	613
Non-Hispanic White [Ref]	77.8 (72.4, 83.2)	78.4 (51.2, 119)	21.0	26.9	35.2	184	224	295	984
Other Hispanic or Other/multi race	78.2 (61.3, 95.0)	80.8 (53.7, 122)	5.11	22.9	35.3	183	224	404	185
Sex									
Female [Ref]	79.1 (72.1, 86.1)	80.5 (50.7, 127)	18.5	25.8	34.0	197	247	338	1,379
Male	75.9 (71.4, 80.5)	77.8 (53.1, 116)	20.5	28.6	36.9	167	209	255	1,088

[a]Geometric mean (95% confidence intervals). [b]Sample size.

Table S3. Sample-weighted multiple regression slopes for urinary 3HPMA and CEMA concentrations (µg/L) among NHANES 2005-2006 non-tobacco users.

**Variable**	**3HPMA Coefficient (95% CI)**[a]	***p***[b]	**CEMA Coefficient (95% CI)**	***p***[b]
Intercept	71.0 (20.1, 122)	<.0001	15.3 (2.67, 27.8)	<.0001
Serum cotinine (ng/mL)	5.65 (-6.49, 17.8)	0.80	3.16 (-0.35, 6.67)	0.007
Creatinine (g/mL)	244207 (207840, 280574)	<.0001	85888 (75090, 96685)	<.0001
Age (yr)				
12-19	-71.5 (-122, -20.7)	0.008	-21.4 (-39.7, -3.08)	0.074
20 - 39	-1.42 (-46.2, 43.4)	0.17	-15.2 (-29.6, -0.86)	0.033
40 - 59 [Ref]	Ref.	.	Ref.	.
≥ 60	-15.2 (-62.3, 32.3)	0.19	23.9 (5.82, 41.9)	0.003
Sex				
Female [Ref]	Ref.	.	Ref.	.
Male	-10.5 (-55.5, 34.4)	0.12	-9.28 (-22.1, 3.55)	0.29
Race/ethnicity				
Mexican American	62.0 (17.6, 106)	0.007	2.55 (-8.66, 13.8)	0.15
Non-Hispanic Black	-37.3 (-93.8, 19.2)	0.067	14.6 (-1.76, 31.0)	0.03
Non-Hispanic White [Ref]	Ref.	.	Ref.	.
Other Hispanic or Other/Multi Race	7.94 (-45.5, 61.4)	0.18	12.6 (-17.5, 42.8)	0.64

[a](95% CI): 95% confidence intervals. [b]*p* value was estimated from identical models where the dependent variable was natural log (ln)-transformed.

Table S4. Sample-weighted multiple regression slopes for urinary 3HPMA and CEMA concentrations (μg/L) among NHANES 2005-2006 exclusive cigarette smokers with serum cotinine > 10 ng/mL.

**Variable**	**3HPMA Coefficient (95% CI)**[a]	***p***	**CEMA Coefficient (95% CI)**	***p***
Intercept	-157 (-357, 43.4)	<.0001	-28.9 (-52.3, -5.50)	<.0001
Creatinine (g/mL)	775000 (538000, 1010000)	<.0001	149000 (121000, 178000)	<.0001
Sex				
Male	-267 (-509, -25.0)	0.29	-31.4 (-64.6, 1.83)	0.51
Female [Ref]	Ref.		Ref.	
Age (yr)				
12-19	-614 (-934, -293)	0.005	-86.4 (-141, -31.5)	0.008
20 - 39	-462 (-629, -296)	0.0005	-87.4 (-131, -43.6)	<.0001
40 – 59 [Ref]	Ref.		Ref.	
≥ 60	-240 (-472, -6.92)	0.03	6.58 (-37.6, 50.7)	0.96
Race/ethnicity				
Non-Hispanic White [Ref]	Ref.		Ref.	
Non-Hispanic Black	-282 (-585, 20.3)	0.31	48.5 (-32.5, 130)	0.22
Mexican American	-130 (-359, 99.4)	0.57	-10.4 (-34.2, 13.3)	0.99
Other Hispanic or Other/multi race	-306 (-583, -28.6)	0.36	-26.7 (-89.6, 36.1)	0.36
Cigarettes smoked per day (5 day)				
Serum cotinine ≤ LOD [Ref]	Ref.		Ref	
1 - 10 CPD (0.5 pack)	712 (582, 842)	< .0001	109 (86.2, 132)	< .0001
11 - 20 CPD (1 pack)	1910 (1620, 2200)	<.0001	250 (206, 294)	<.0001
21 - 30 CPD (1.5 packs)	2120 (1480, 2750)	<.0001	344 (245, 492)	<.0001
>30 CPD (>1.5 packs)	2520 (1720, 3320)	0.0007	368 (245, 492)	<.0001

CPD: Cigarettes smoked per day. [a](95% CI): 95% confidence intervals.
